# Structural Determination of Functional Units of the Nucleotide Binding Domain (NBD94) of the Reticulocyte Binding Protein Py235 of *Plasmodium yoelii*

**DOI:** 10.1371/journal.pone.0009146

**Published:** 2010-02-10

**Authors:** Ardina Grüber, Malathy S. S. Manimekalai, Asha M. Balakrishna, Cornelia Hunke, Jeyaraman Jeyakanthan, Peter R. Preiser, Gerhard Grüber

**Affiliations:** 1 School of Biological Sciences, Nanyang Technological University, Singapore, Singapore; 2 National Synchrotron Radiation Research Center, Hsinchu, Taiwan; Bernhard Nocht Institute for Tropical Medicine, Germany

## Abstract

**Background:**

Invasion of the red blood cells (RBC) by the merozoite of malaria parasites involves a large number of receptor ligand interactions. The reticulocyte binding protein homologue family (RH) plays an important role in erythrocyte recognition as well as virulence. Recently, it has been shown that members of RH in addition to receptor binding may also have a role as ATP/ADP sensor. A 94 kDa region named Nucleotide-Binding Domain 94 (NBD94) of *Plasmodium yoelii* YM, representative of the putative nucleotide binding region of RH, has been demonstrated to bind ATP and ADP selectively. Binding of ATP or ADP induced nucleotide-dependent structural changes in the C-terminal hinge-region of NBD94, and directly impacted on the RBC binding ability of RH.

**Methodology/Principal Findings:**

In order to find the smallest structural unit, able to bind nucleotides, and its coupling module, the hinge region, three truncated domains of NBD94 have been generated, termed NBD94_444–547_, NBD94_566–663_ and NBD94_674–793_, respectively. Using fluorescence correlation spectroscopy NBD94_444–547_ has been identified to form the smallest nucleotide binding segment, sensitive for ATP and ADP, which became inhibited by 4-Chloro-7-nitrobenzofurazan. The shape of NBD94_444–547_ in solution was calculated from small-angle X-ray scattering data, revealing an elongated molecule, comprised of two globular domains, connected by a spiral segment of about 73.1 Å in length. The high quality of the constructs, forming the hinge-region, NBD94_566–663_ and NBD94_674–793_ enabled to determine the first crystallographic and solution structure, respectively. The crystal structure of NBD94_566–663_ consists of two helices with 97.8 Å and 48.6 Å in length, linked by a loop. By comparison, the low resolution structure of NBD94_674–793_ in solution represents a chair–like shape with three architectural segments.

**Conclusions:**

These structures give the first insight into how nucleotide binding impacts on the overall structure of RH and demonstrates the potential use of this region as a novel drug target.

## Introduction

**Figure 1:**
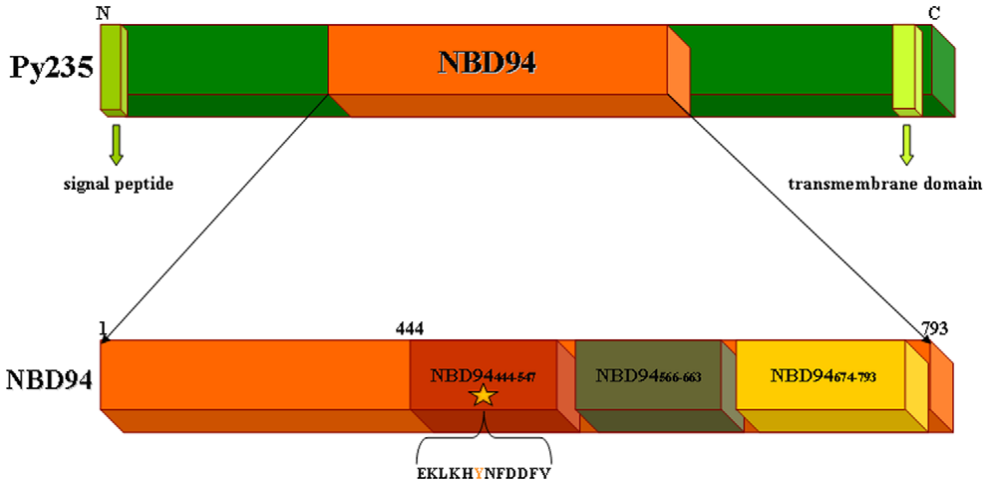
Domain features of Py235 of *P. yoelii*. Three truncated constructs, called NBD94_444–547_ and NBD94_566–663_ as well as NBD94_674–793_, both described to belong to the C-terminal hinge region [27], have been generated. NBD94_444–547_ includes the peptide _488_EKLKHYNFDDFVK_500_, which has been shown to covalently bind the nucleotide-analogue 8-N_3_-3′-biotinyl-ATP [27], is highlighted as a star.

Malaria continues to be one of the major public health problems for mankind; it is the transmissible disease with the greatest morbidity around the world [1]. The complex life cycle of the protozoan parasite is characterized by distinct invasive forms of the sporozoite and merozoite that invade hepatocytes and erythrocytes in the vertebrate host, respectively, and the ookinetes inside the insect vector that penetrates the mosquito midgut epithelium [1]–[6]. Invasion of red blood cells (RBC) by the merozoite and the subsequent cyclical replication of the parasite is the cause of malaria associated pathology. Multiple merozoite protein families are implicated in the invasion of RBCs, including the erythrocyte binding proteins (EBPs) and the reticulocyte binding protein homologues (RH), which bind to different RBC membrane receptors [1], [6]–[16]. Little is known about how the large RH transmembrane proteins mediate their function during erythrocyte invasion, but a crucial step appears to be the proteolytic cleavage during the invasion process [14], [17]. Members of RH have been identified in all *plasmodium* species so far analyzed indicating the conserved function and importance of this protein family to the malaria parasite [1]. The *P. yoelii* RH protein, termed Py235 (235 kDa in mass), has been shown to be a potential virulence factor that allows the parasite to invade a wider range of host erythrocytes [18]–[20]. Py235 is also involved in clonal phenotypic variation of merozoites [21], enabling the parasite to evade immune responses and adapt to changes in the host environment during the invasion step [22]. Previous studies in *P. vivax* has indicated that RH may have an initial sensing role preceding and possibly enabling the subsequent interaction of the EBP member with its corresponding receptor [23]. Besides multiple receptor ligand interaction between the merozoite and the RBC, the effects of other intra and/or extra cellular molecules like ATP on invasion have been discussed [24]–[27]. ATP, released by erythrocytes under normal physiological conditions, has recently been shown to serve as a signalling molecule regulating vascular tone [24]–[26] and ATP receptors have been implicated in a number of signalling pathways [28], [29]. Mechanical deformation of erythrocytes as encountered in capillaries forming the microvasculature lead to increased ATP release [30]. Importantly, intracellular ATP is a requirement for merozoite invasion [31]–[34] with erythrocytes that have been depleted of ATP being refractory to invasion. These findings suggest that it would be advantageous for merozoites to sense the intracellular ATP level of the erythrocyte. In this context a 94 kDa domain of Py235 of *P. yoelii*, which is highly conserved amongst the RBLs and called the nucleotide-binding domain (NBD94), has been found to selectively bind ATP and ADP. The amino acid sequence _488_EKLKHYNFDDFVK_500_ in NBD94 has been identified as nucleotide-binding region as shown by photoaffinity labeling of the nucleotide-analogue 8-N_3_-3′-biotinyl-ATP (Fig. 1 [27]). The preference of MgATP over MgADP recognition is associated with specific structural alterations in the C-terminal domain of NBD94 as depicted by spectroscopic comparison of NBD94 and its C-terminal truncated form, NBD94_1–550_, in which no nucleotide-dependent alteration could be observed [27]. The nucleotide effect in the recombinant protein is confirmed by a strong binding of Py235 to RBCs in the presence of MgATP which is significantly lower in the presence of MgADP or the absence of nucleotides [27]. Based on these traits and the absence of significant ATPase activity of NBD94, this domain was suggested to serve as an ATP/ADP sensor during the invasion process [27]. However, the recombinant NBD94 protein is prone to degradation over a period of time. Whether degradation occurs through autolysis or proteolytic cleavage, as described for a series of proteolytic cleavage events of both the EBP and RH protein families during invasion, resulting in 9 to 13 kDa fragments, is unclear [35], [36].

In many cases a protein fragment obtained by cleavage of the full length protein or by expression of part of the protein can retain a functional domain. This is particularly relevant in drug designing. In order to get insight into the functional regions of NBD94 and to define the smallest segment, still able to bind ATP/ADP, three truncated constructs, called NBD94_444–547_, including the 8-N_3_-3′-biotinyl-ATP binding sequence, as well as NBD94_566–663_ and NBD94_674–793_, described to belong to the C-terminal hinge region and to couple the nucleotide-binding event [27], have been generated (Fig. 1) and purified to homogeneity. As demonstrated by fluorescence correlation spectroscopy (FCS), NBD94_444–547_ forms the smallest ATP/ADP-binding segment, which becomes inhibited by the ATPase/-synthase inhibitor 4-Chloro-7-nitrobenzofurazan (NBD-Cl). The high quality of all proteins formed the platform to solve the first low resolution structure of NBD94_444–547_, and NBD94_674–793_ in solution and the first crystallographic structure of NBD94_566–663_. The structural features of this ensemble will be discussed in the light of coupling of nucleotide-binding with structural rearrangements in the hinge region, followed by downstream effects, enabling the parasite to continue the invasion process.

## Results

### Production and Purification of Truncated Forms of NBD94 of *Plasmodium yoelii*

**Figure 2:**
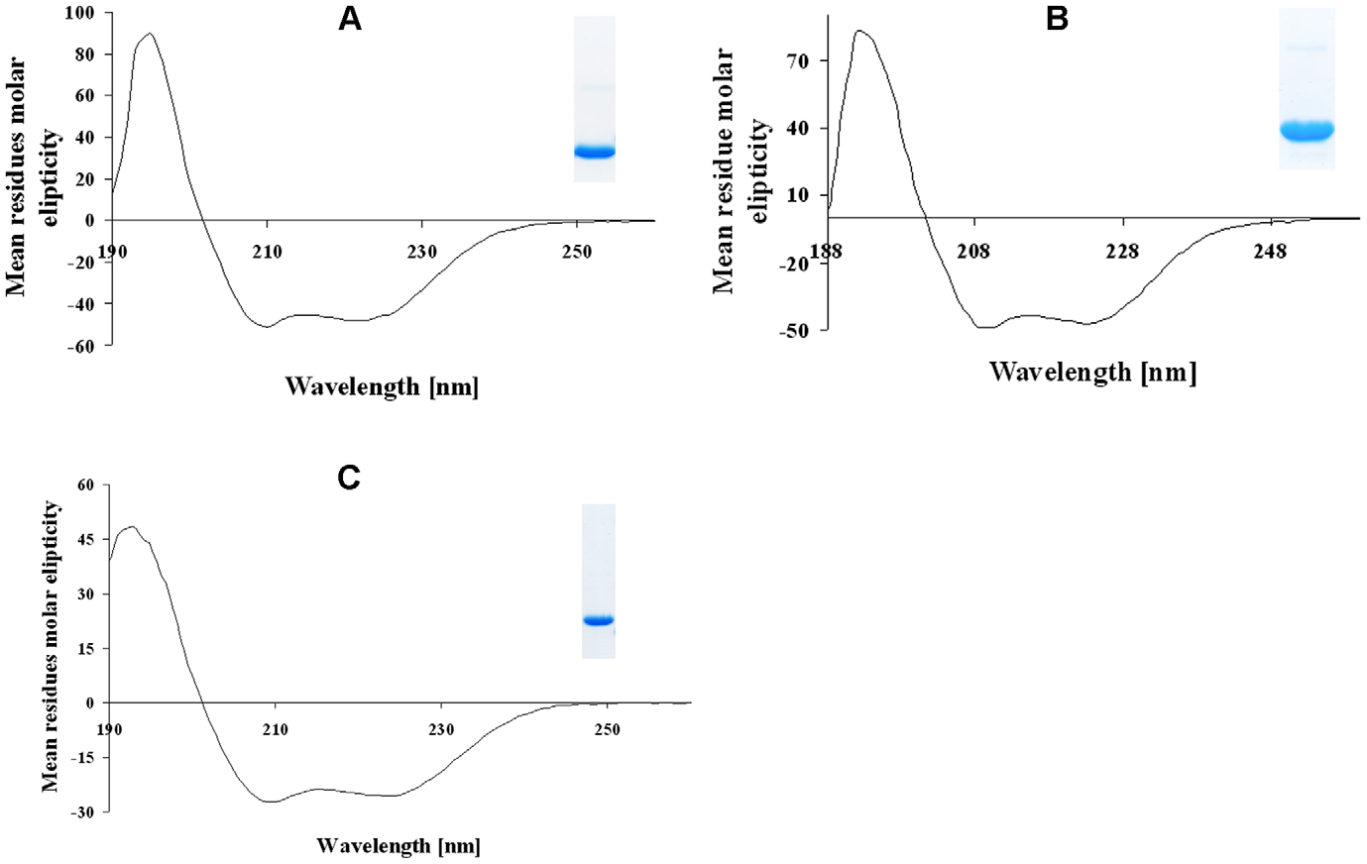
Circular dichroism (CD) spectroscopy of the recombinant proteins. (*A–C*), Far-UV CD spectra of NBD94_444–547_ (*A*), NBD94_566–663_ (*B*) as well as NBD94_674–793_ (*C*), respectively. The SDS-gel in the insets show a sample of the corresponding purified constructs NBD94_444–547_, NBD94_566–663_ and NBD94_674–793_.

In order to understand the events of nucleotide-binding in NBD94 and its concerted structural alteration(s) in the C-terminal hinge region, as well as to determine the smallest segment of NBD94, still able to bind nucleotide, and thereby forming an attractive target for therapeutic agents, the truncated forms NBD94_444–547_, NBD94_566–663_ and NBD94_674–793_ have been generated (Fig. 1), in which predicted α-helical structures have been taken into account (see below). The SDS-PAGE of the produced recombinant NBD94_444–547_, NBD94_566–663_ and NBD94_674–793_ of *Plasmodium yoelii* revealed a prominent band of 13.2 kDa, 12.8 kDa and 15.2 kDa, respectively, which was found entirely within the soluble fraction. A Ni^2+^-NTA resin column and an imidazole-gradient were used to separate NBD94_444–547_, NBD94_566–663_ and NBD94_674–793_, respectively, from the main contaminating proteins. The protein NBD94_444–547_, NBD94_566–663_ and NBD94_674–793_, respectively, eluting at 125–300 mM imidazole were collected, concentrated and subsequently applied to a size exclusion column (Superdex 75 HR 10/30 column). Analysis of the isolated protein by SDS-PAGE revealed the high purity of NBD94_444–547_, NBD94_566–663_ and NBD94_674–793_ (Fig. 2A–C).

The secondary structures of these proteins were determined from circular dichroism spectra, measured between 185–260 nm (Fig. 2A–C). The CD-spectra of NBD94_444–547_, NBD94_566–663_ and NBD94_674–793_ show that all proteins are mainly α-helical, as reflected by its minima at 208 and 222 nm and as predicted from its amino acid sequence. The α-helical content of NBD94_444–547_, NBD94_566–663_ and NBD94_674–793_ is determined to be 83%, 86% and 69%, respectively, congruent to the values determined for the entire NBD94 and indicating the correct secondary structure of the recombinant truncated proteins generated, as well as reflecting a proper selection of stable constructs. The molar ellipticity values at 208 nm and at 222 nm of NBD94_444–547_, NBD94_566–663_ and NBD94_674–793_ are in a ratio of 0.94, 0.99 and 0.97, respectively.

### Shape Determination of NBD94_444–547_ and NBD94_674–793_ in Solution

**Figure 3:**
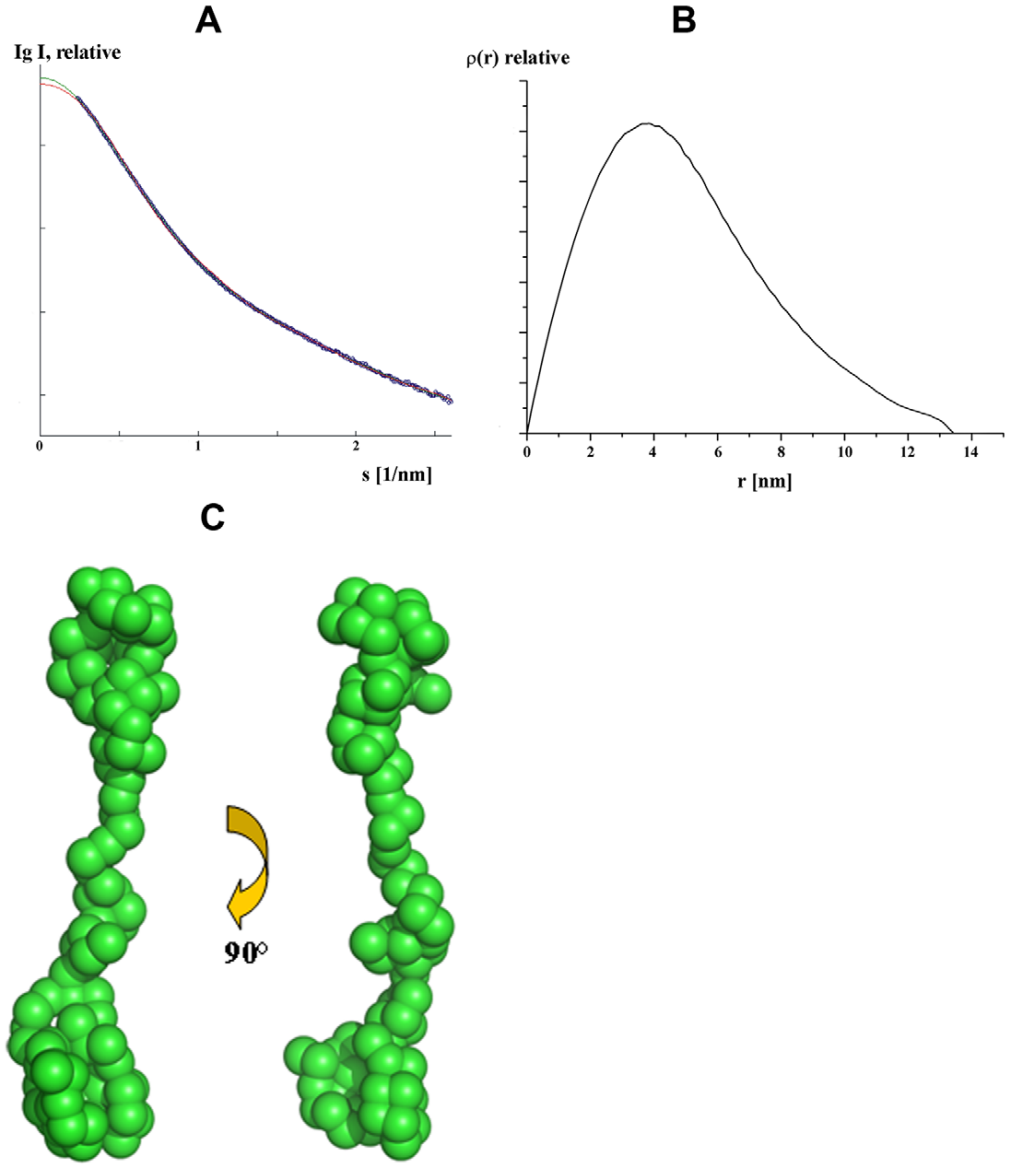
Small-angle X-ray scattering data of NBD94_444-547_ of *P. yoelii*. (*A*) Experimental scattering data (o) and the fitting curves (—; green: experimental, red: calculated from *ab initio* model) for NBD94_444–547_ of *P. yoelii*. (*B*) The distance distribution function of the same protein. (*C*) Low resolution structure of NBD94_444–547_ in solution determined from SAXS data. The two shapes are rotated clockwise by around 90° along the Y-axis.

**Figure 4:**
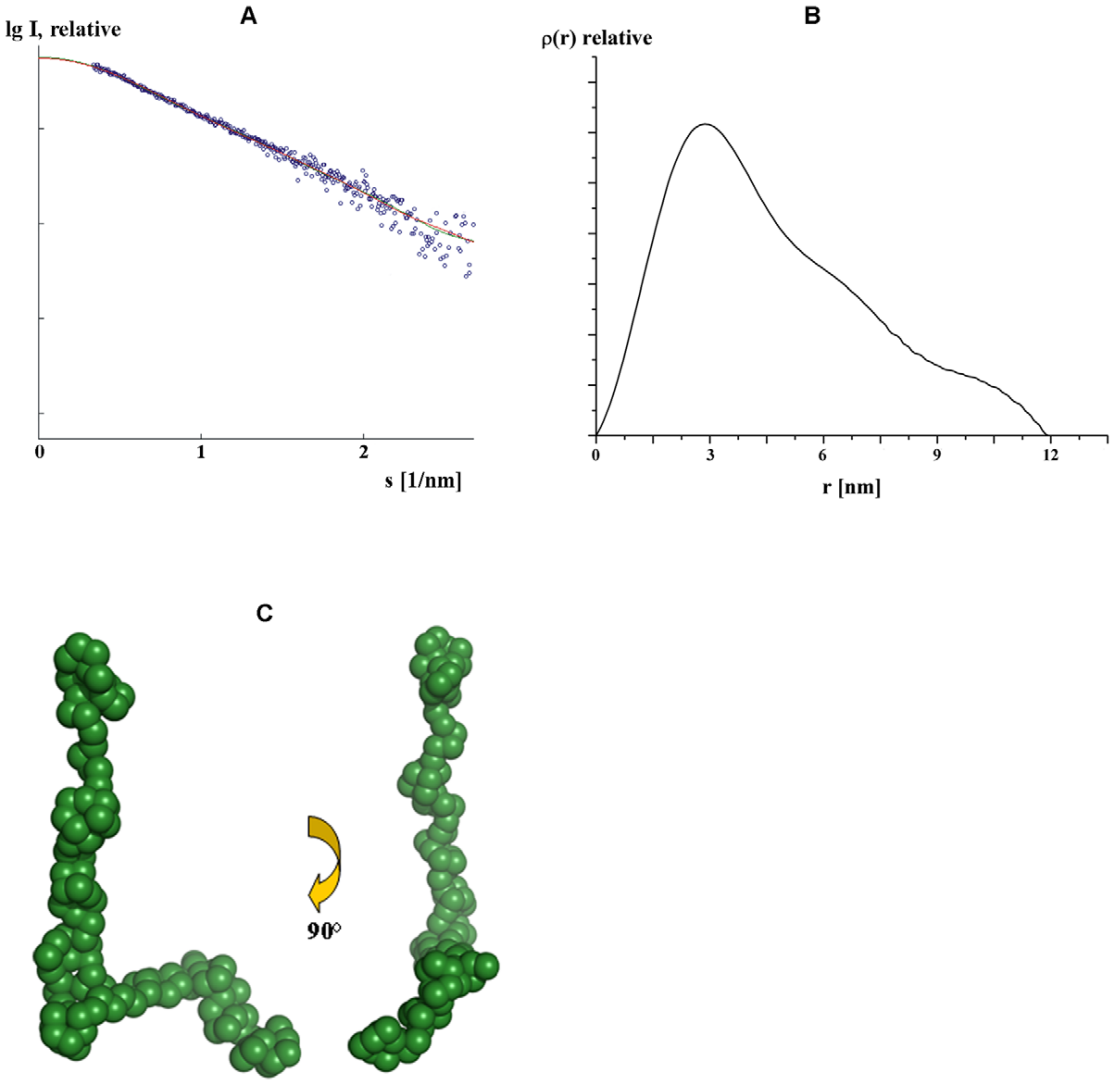
SAXS data of NBD94_674–793_. (*A*) Experimental (o) and the fitting scattering curves (—; green: experimental, red: calculated from *ab initio* model) and (*B*) the distance distribution function for NBD94_674–793_ of *P. yoelii*. (*C*) Shape of NBD94_674–793_ in solution determined from SAXS data. The two low resolution structures are rotated clockwise by around 90° along the Y-axis.

The high purity allowed small-angle X-ray scattering (SAXS) experiments to be performed, with the aim to determine the first low resolution structures of the nucleotide-binding segment NBD94_444–547_ and NBD94_674–793_ in solution. SAXS patterns from solutions of both proteins were recorded as described in Materials and Methods to yield the final composite scattering curves in figure 3A and 4A, respectively, that both proteins are monodispersed in solution. The radius of gyration *R_g_* of NBD94_444–547_ is 33.4±1 Å and the maximum dimension *D_max_* of the protein is 134±2 Å (Fig. 3B). The gross structure of NBD94_444–547_ was restored *ab initio* from the scattering pattern in figure 3A using the dummy residues modeling program GASBOR [37], which fitted well to the experimental data in the entire scattering range (a typical fit displayed in figure 3A, curve 2, has the discrepancy χ = 1.28). Ten independent reconstructions yielded reproducible models and the average model and the most probable model are displayed in figure 3C. NBD94_444–547_ appears as an elongated molecule with a length of 134 Å, composed of two more globular domains and a spiral-like segment of about 73.1 Å in length between both domains.

By comparison, the radius of gyration *R_g_* of NBD94_674–793_ is 22.2±1 Å nm and the maximum dimension *D_max_* of the protein is 118±2 Å (Fig. 4B), respectively, suggesting that the protein is rather elongated. The shoulders at larger intraparticle distances of about 70 Å and 100 Å indicate that the molecule consists of three distinct domains. Like for NBD94_444–547_ the shape of NBD94_674–793_ was determined *ab initio* from the scattering pattern in figure 4A using the program GASBOR [37]. The experimental data fitted well with the calculated data, reflected by an χ value of 1.03 (Fig. 4A, curve (—; red)). The *ab initio* modeling produced a chair–like shape (Fig. 4C) with three domains of 96 Å, 35 Å and 20 Å in length. When rotating the molecule by 90°, the longer domain has a spiral feature and the lower 20 Å long domain turns away from the middle one by 104.5°.

### Nucleotide-Binding Determined by Fluorescence Correlation Spectroscopy

**Figure 5:**
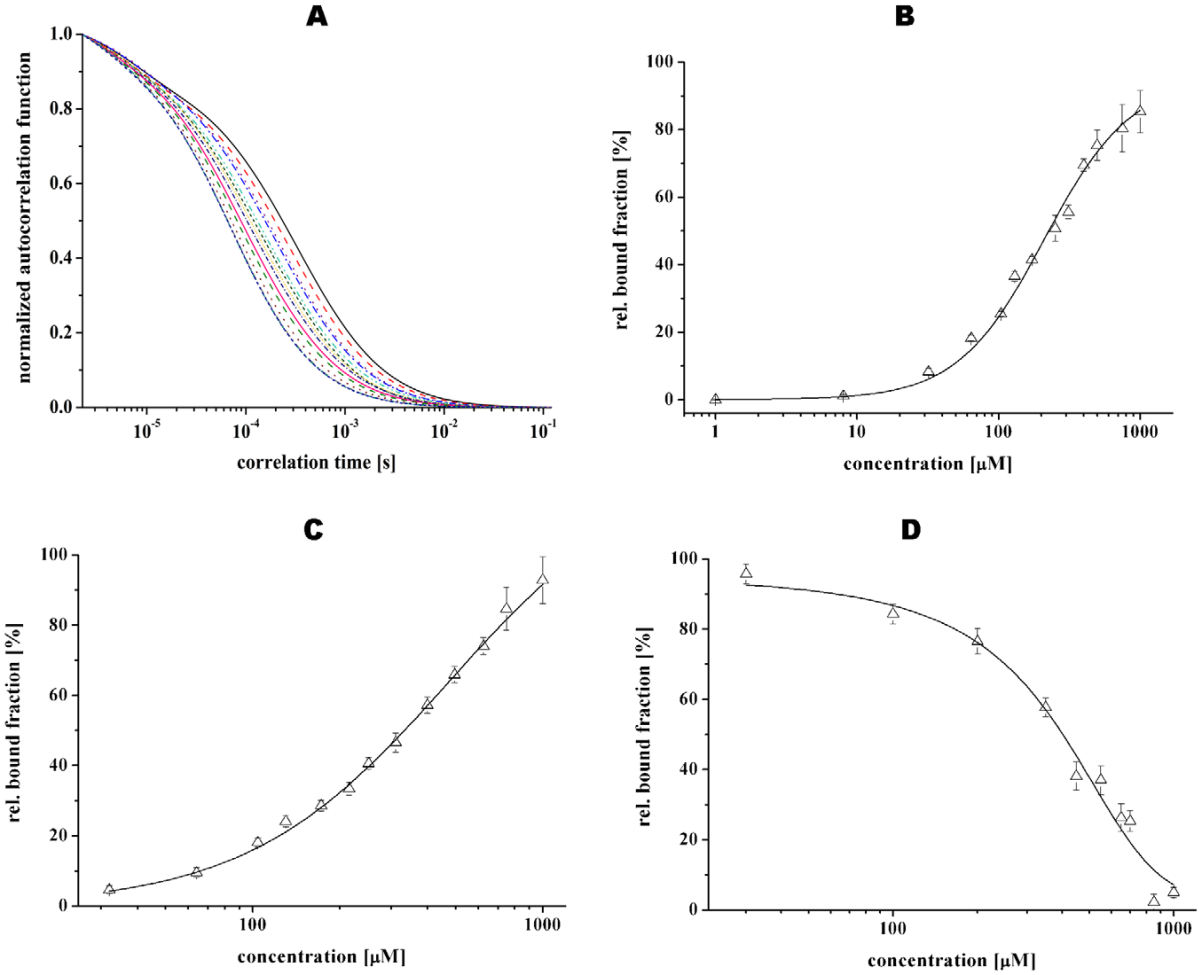
Binding traits of NBD94_444–547_ to fluorescently labeled MgATP ATTO-647N. (*A*) Normalized autocorrelation functions of MgATP ATTO-647N (*B*) obtained by increasing the quantity of NBD94_444–547_ (increased protein concentration from left to right). (*C*) Binding of NBD94_444–547_ to MgADP ATTO-647N. The nucleotide analogue is displayed as relative bound fraction versus protein concentration. The best fit to titration curve A and B are shown as a non-linear, logistic curve fit. (*D*) Influence of NBD-Cl to MgATP ATTO-647N binding traits of NBD94_444–547_. The best fits at titration curves of supplementary figure 1B are shown as a pharmacological dose-response curve with variable Hill slope.

The proper structural folding and structure formation of NBD94_444–547_ enabled us to study the ability of this protein to bind nucleotides by fluorescence correlation spectroscopy using fluorescent ATP and ADP derivatives ATP ATTO-647N and ADP ATTO-647N, respectively. As a reference, the mean count rate per Cyanine 5 (Cy5) fluorophore was determined to be 32.5±0.4 kHz. Compared to Cy5, the value of ATP ATTO-647N was determined to be 27.9±0.8 kHz and 51.3±3.5 kHz for ADP ATTO-647N. Fitting the autocorrelation functions resulted in characteristic times of diffusion τ_D_ = 50±1.1 µs for Cy5, τ_D_ = 70.2±1.3 µs for ATP ATTO-647N and τ_D_ = 68.9±2.2 µs for ADP ATTO-647N. The autocorrelation curves of the fluorescent ATP analogue for ATP ATTO-647N and ADP ATTO-647N in the absence and presence of increased concentrations of NBD94_444–547_ are show in figure 5A and in supplementary figure S1A, respectively. The increase of the mean diffusion time τ*_D_* was due to the increase in the mass of the diffusing particle, when fluorescently labelled nucleotide bound to NBD94_444–547_, which is apparent in the displayed autocorrelation curves with increased protein concentrations from left to right. A binding constant (*K_d_*) of 228±2.3 µM for MgATP ATTO-647N and 331±1.8 µM for MgADP ATTO-647N bound to NBD94_444–547_ was determined (Fig. 5B, C). By contrast no nucleotide-binding could be observed in the constructs NBD94_566–663_ and NBD94_674–793_.

Most recently, we observed that 4-Chloro-7-nitrobenzofurazan (NBD-Cl) is a potent inhibitor of nucleotide-binding in the entire NBD94 [27]. We tested, whether MgATP ATTO-647N-binding of the minimal nucleotide-core domain, NBD94_444–547_, becomes inhibited by NBD-Cl. The plotted autocorrelation functions in supplementary figure S1B show a change of the diffusion time due to an increase of the concentration of NBD-Cl from right to left for MgATP ATTO-647N. The calculated bound fraction for increasing inhibitor concentrations were plotted to determine the effect of nucleotide binding. The interaction of MgATP ATTO-647N to NBD94_444–547_ showed an IC_50_ value of 400±3 µM (Fig. 5D). A similar effect of NBD-Cl-inhibition has been observed for NBD94_444–547_-ADP-ATTO-647N formation (data not shown).

### Crystallographic Structure of NBD94_566–663_

The second domain, NBD94_566–663_, covering the amino acids close to the nucleotide binding segment NBD94_444–547_, has been crystallized by vapor diffusion using 2-methyl-2,4 pentanediol as a precipitant in a form suitable for X-ray diffraction analysis. This success was due to controlling the rate of vapor diffusion via the introduction of an oil barrier over the reservoir of a vapor-diffusion trial. The two different oils, paraffin and silicone, were applied as barriers, separately and also as mixtures of these two oils. Paraffin oil enabled the increase of crystal size (Supplementary Figure S2A and S2B) as well as improvement in resolution. The native crystal diffracted to 4.1 Å while the SeMet crystals diffracted to 3.9 Å at peak wavelength. A MAD dataset was collected which was indexed, integrated and scaled using the *HKL*-2000 [38] package. The data collection statistics are given in Table 1. Both the native and the SeMet derivative, crystallizes in the hexagonal space group P6_3_22 with three molecules in the asymmetrical unit and a solvent content and *V*_M_ value of 35.44% and 1.9 Å^3^ per Dalton, respectively [39]. The data collection statistics are given in Table 1.

**Table 1:** Statistics of crystallographic data collection for NBD94_566–663._

	Se-derivative	Peak	Edge	Remote
*Data collection statistics*	Wavelength (Å)	0.97898	0.97936	0.96412
	Space group	P6_3_22	P6_3_22	P6_3_22
	Unit cell parameters	a = b = 70.22 Åc = 193.22 Å α = β = 90° γ = 120°		
	Resolution range (Å)	30–3.9	30–4.0	30-4.15
	Number of unique reflections	2936	2762	2789
	I/σ^a^	13.71 (3.69)	23.32 (2.98)	19.64 (3.75)
	Completeness (%)	95.6 (97.1)	85.6 (86.9)	81.6 (76.9)
	R merge^b^ (%)	10.2 (39.3)	6.8 (42.9)	7.0 (34.8)
	Multiplicity	15.4 (12.4)	15.0 (12.1)	14.4 (11.6)
*Refinement statistics*	Resolution range (Å)	30-4.0		
	R factor^c^ (%)	33.90		
	R free^d^ (%)	37.32		
	R factor (all reflections)	34.86		
*Ramachandran statistics*	Most favored (%)	81.1		
	Additionally allowed (%)	17.3		
	Generously (%)	1.6		
*R.M.S. deviations*	Bond lengths (Å)	0.004		
	Bond angles (°)	0.839		
*Mean atomic B values*	Overall	64.57		
	Wilson plot	81.82		

a Values in parentheses refer to the corresponding values of the highest resolution shell; for peak (4.04-3.9), edge (4.14-4.0) and remote (4.14-4.0).

b R_merge_ = ΣΣ_i_|I_h−_I_hi_|/ΣΣ_i_ I_h_, where I_h_ is the mean intensity for reflection h.

c R-factor = Σ||F_O_|−|F_C_||/Σ|F_O_|, where F_O_ and F_C_ are measured and calculated structure factors, respectively.

d R-free = Σ||F_O_|−|F_C_||/Σ|F_O_|, calculated from 10% of the reflections selected randomly and omitted from the refinement process.

**Figure 6:**
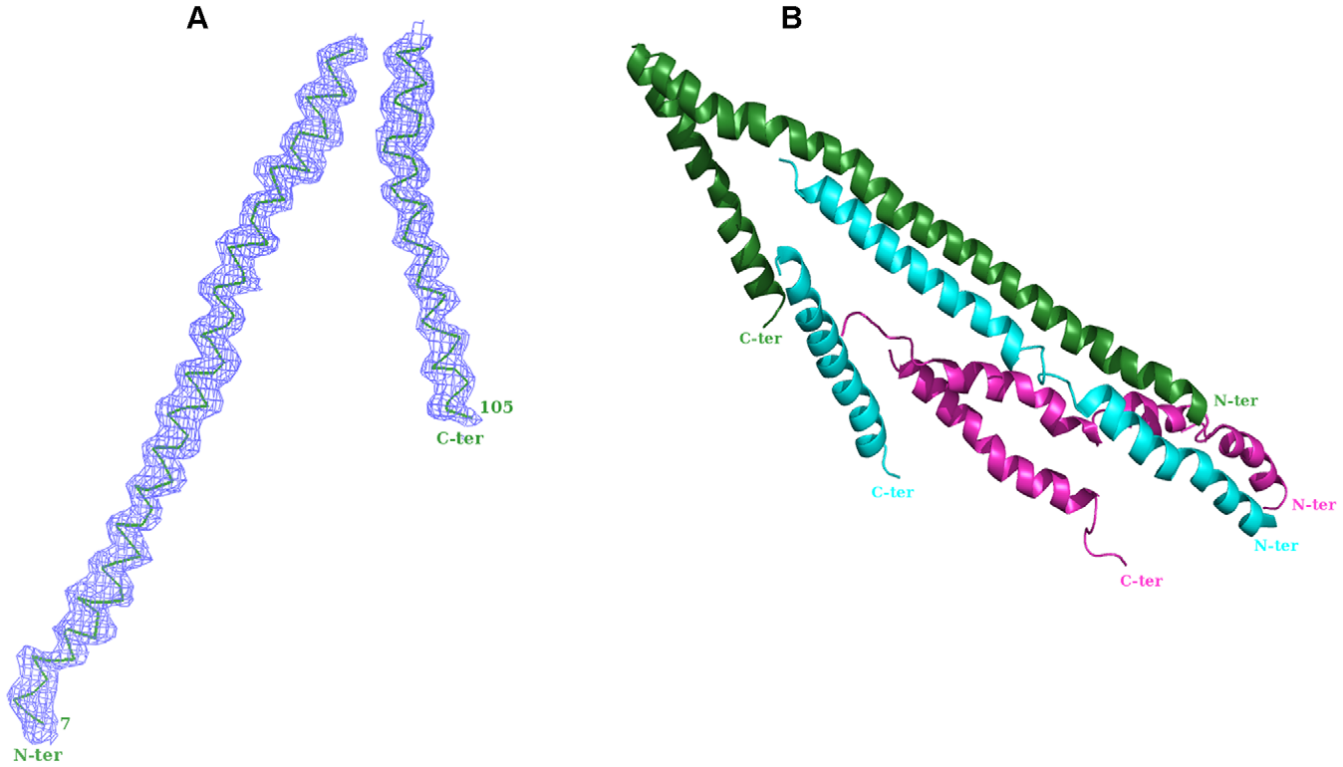
Electron density map and crystallographic structure of NBD94_566–663_ of *P. yoelii*. (*A*), The NBD94_566–663_ molecule with its electron density map (2Fo-Fc at 1.5σ level) (*blue*). (*B*), Crystallographic structure of the A (*green*) B (*blue*) and C (*magenta*) chains of NBD94_566–663_. (C)

Both MAD and SAD techniques for structure solution were tried. Various programs like, SHELX [40], SOLVE [41], CNS [42] and CCP4 [43] were used to identify the heavy atom sites (Se). Reasonable statistics and good initial map could be achieved with the SAD technique and using the help of SHELX program [40]. The resolution cut-off used for Se site identification is 4.4 Å and the correlation co-efficient stood at 39.51% with the Pseudo-free CC of 71.47%. Peak wavelength (0.97898 Å) data was used for the SAD technique in which 12 out of 18 Se sites (6 SeMet in each molecule) could be identified by SHELXD [44] that were further refined by SHELXE [45]. These 12 Se-sites were used to phase the structure factor with SHELXE and the resulting electron densities were improved by solvent flattening with SOLOMON and density modification by DM from the CCP4 package and RESOLVE [46]. The helical region could be readily identified from the initial map and the model of the NBD94_566–663_ was built manually using the program COOT [47]. Several cycles of manual building and fitting were carried out by COOT in combination with restrained refinement using REFMAC5 [48] of the CCP4 suite, keeping the temperature factor at overall. Each time a simulated annealing omit map was calculated and was used for model building (Supplementary Figure S3). The electron density map for the main chain atoms is excellent showing the typical sausage like features for the helical regions (Fig. 6A), whereas for the side chain atoms no visible densities could be identified. Se positions from the anomalous map were used to trace the chains. The final refined model has an R-factor of 33.9% and a R-free of 37.32% with good stereochemistry as can be inferred from the Ramachandran plot statistics given by PROCHECK [49]. The detailed summary of the refinement statistics is given in Table 1. The final electron density map for the structure shows good density for most of the backbone residues except for the residues 1–6 and 73–75 in chain A (Fig. 6A), 1–12 and 73–75 in chain B and 1–13, 31–33 and 73–75 in chain C, respectively (Fig. 6B).

The structure of the NBD94_566–663_ molecule consists of two helices that are linked by a loop, which is not visible in the electron density map. The length of the long and short helix is 97.8 Å and 48.6 Å, which are formed by 65 and 30 residues, respectively (Fig. 6B). Amino acids Gly61 and Ser85 of the N- and C-terminal helix, respectively, are in a very close proximity of 9.85 Å. Due to the kinks from residues Tyr60-Lys62 and Ser85-Glu87 both helices spread apart by an angle of 40.2°, thereby forming a hinge-like feature. The total number of amino acid residues is 107 that includes the N-terminal His-tag residues. The longer helix of chain C in the asymmetric unit is highly distorted when compared to the other molecules (Fig. 6B). Slight kinks could be noted in the long helix from residues Val20-Lys22 and Tyr60-Lys62 in chain A. Whereby in chain B, the residues Asp24-Lys26 show slight helical distortion but for residues Leu37-Lys47 the helix is deviated more. The shorter helices of all the three molecules do show some amount of distortion but is less when compared to the long helices, wherein residues Ser85-Glu87 in chain A, Glu80-Met82 in chain B and Tyr89-Lys91 in chain C show notable deviations.

## Discussion

The mechanism by which a merozoite recognizes a suitable host cell is mediated by a cascade of receptor ligand interactions. In addition to the availability of the appropriate receptors, intracellular ATP plays an important role in determining if erythrocytes are suitable for merozoite invasion [24]–[26]. In erythrocyte binding assays (EBA) it has been demonstrated that Py235 of *P. yoelii* binds strongly to erythrocytes in the presence of ATP, whereby weak interactions have been found in the presence of ADP or the absence of nucleotides [27]. The ATP/ADP modulation of Py235-receptor binding suggested a nucleotide-dependent rearrangement, making the binding domain of Py235 more accessible. Such a nucleotide induced change has been observed in the 94 kDa nucleotide-binding domain, NBD94, of Py235, in which ATP-binding causes alterations in the C-terminal hinge region [27]. The recombinant NBD94_444–547_ is identified as the smallest segment of NBD94 still able to bind nucleotides with a preference of ATP- over the ADP analogue, important for sensing the signal for receptor binding of Py235. NBD94_444–547_ includes the _488_EKLKHYNFDDFVK_500_ peptide, observed to bind the nucleotide-analogue 8-N_3_-3′-biotinyl-ATP [27]. Tyr493 is the only Tyr residue of this segment and thereby the candidate for covalently binding the potent inhibitor NBD-Cl, which was predicted to act as a nucleotide analogue in F_1_F_O_ ATP synthases [50]. NBD-Cl interacts with the phenolic oxygen of Tyr331 in the catalytic β subunit of bovine F_1_F_O_ ATP synthases and appears to act by preventing the nucleotide empty β subunit from converting to a productive nucleotide-binding state [51]–[53]. The hydrated NBD94_444–547_ is a 134 Å long molecule, comprised of two globular segments, connected by a spiral region of about 73.1 Å in length with an α-helical content of 83%. The *Θ_222_/Θ_208_* ratio of 0.94 indicates that many of the residues in NBD94_444–547_ are in close neighborhood, since non-interacting helices typically give ratios of around 0.8. As ATP/ADP-binding in NBD94 [27] and NBD94_444–547_ is shown to be inhibited by NBD-Cl, proposed to interact with Tyr493, the elongated NBD94_444–547_ becomes a new target for modified or novel classes of ATP agents.

**Figure 7:**
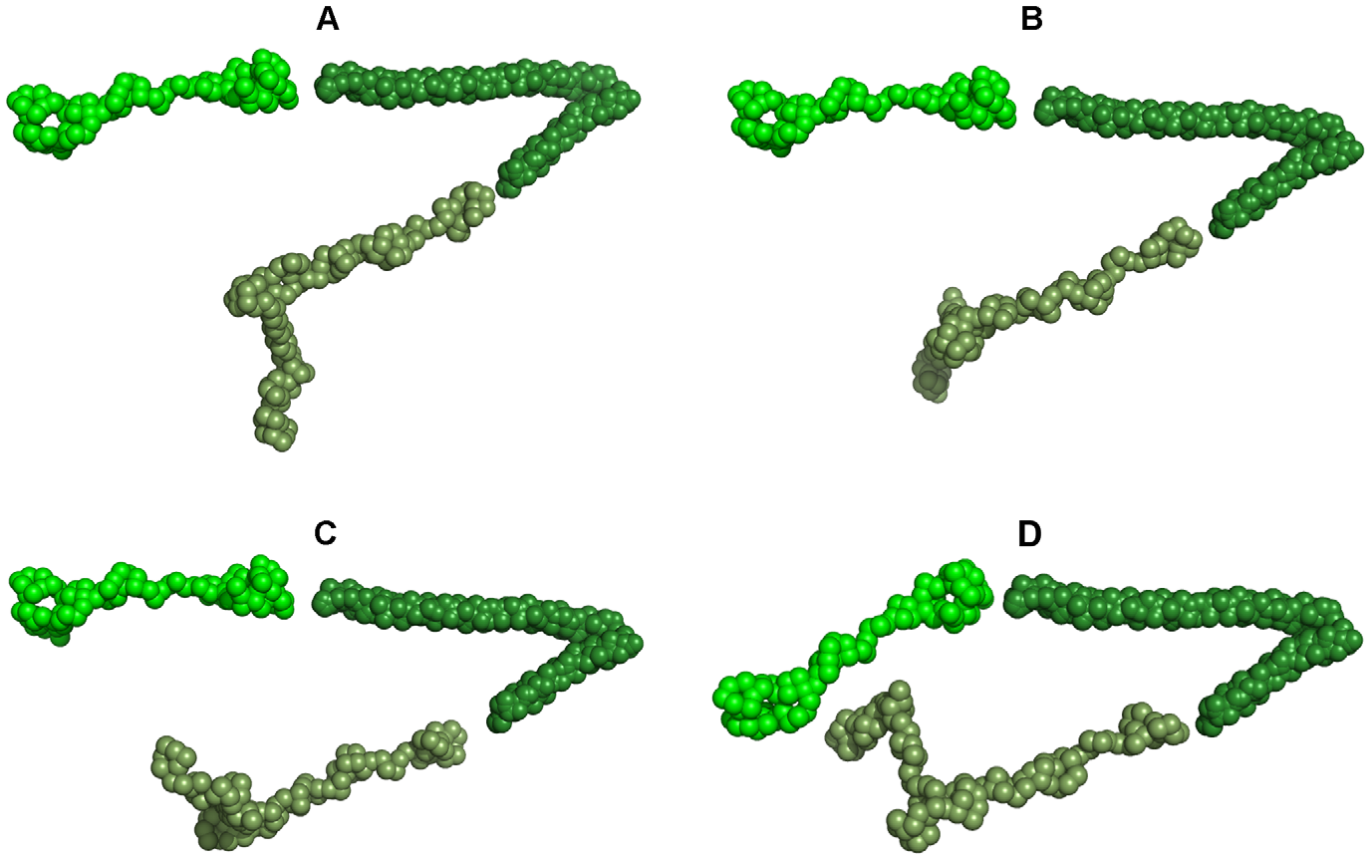
Hypothesized arrangements of the three NBD94 segments. (*A–C*) A gallery of possible arrangements of the nucleotide-binding region NBD94_444–547_ (*green*), the crystallographic NBD94_566–663_ structure (*dark green*) and the very C-terminal structure of NBD94_674–793_ (*olive*), in which the N-terminal helix NBD94_566–663_ is linked to the N-terminal NBD94_444–547_ segment, while the C-terminal helix of NBD94_566–663_ is linked to the very C-terminal NBD94_674–793_. (D) An arrangement in which NBD94_444–547_ and NBD94_674–793_ are in close proximity.

Recently, we proposed that the ATP-binding event in NBD94 triggers a conformation rearrangement within the protein, going along with alterations in Py235 and thereby changing the binding strength of this reticulocyte binding-like protein to the red blood cell, as reflected by erythrocyte binding assays performed with Py235 of *P. yoelii* [27]. From nucleotide-dependent CD-spectroscopy data of NBD94 and its C-terminal truncated form NBD94_1–550_ it was also concluded that a hinge-like region in the C-terminal region of NBD94 may mediate the nucleotide-binding with further down stream events [27]. The NBD94_566–663_ molecule with its long N-terminal and shorter C-terminal α helices, linked by a flexible loop, shows a hinge-like arrangement (Fig. 6B). In the crystallographic structure, the residues Gly61 and Ser85 of the N- and C-terminal helix, respectively, are in a very close proximity of 9.8 Å and the very N- and C-terminal amino acids of NBD94_566–663_ spread apart by an angle of 40.2°. This structural feature of NBD94_566–663_ enables this protein to move either like a hinge or like a rattle up and down, thereby transmitting ATP-ADP modulation in NBD94_444–547_ with up- and down movements in NBD94_566–663_, which have to be coupled to the α-helical and chair-like NBD94_674–793_ fragment of the C-terminal region of NBD94, representing an elongated protein with three subdomains of 96 Å, 35 Å and 20 Å in length. Different orientation of the protein ensemble relative to each other have been speculated, in which the N-terminal segment NBD94_444–547_ is linked to the N-terminal helix of NBD94_566–663_ and the C-terminal segment NBD94_674–793_ is oriented to the C-terminal helix of NBD94_566–663_ (Fig. 7A–C). In all hypothesized models, the structure of the NBD94_566–663_ molecule forms the middle element, able to mediate the sensing of ATP-ADP binding in NBD94_444–547_ with concerted conformational changes in NBD94_566–663_ and NBD94_674–793_, whose subdomains might undergo structural rearrangements, transferred to down-stream events in NBD94, thereby facilitating the linkage of nucleotide signaling and Py235 binding to the blood cell. An alternative prediction is presented in figure 7D revealing the possibility of a direct NBD94_444–547_ and NBD9_674–793_ contact, in which NDB94_566–663_ presents a linking element. The increased nucleotide affinity in presence of the C-terminal domain of NBD94 determined recently [27] may hint at such an assembly.

In summary, the data presented demonstrate that NBD94 can be divided into a nucleotide-binding segment, NBD94_444–547_, existing in solution as an elongated molecule. The first crystallographic and solution structure of NBD94_566–663_ and the C-terminal domain NBD94_674–793_ of the C-terminal hinge region in NBD94 provide the structural basis towards a better understanding of a concerted interaction of the protein ensemble in NBD94, which is triggered by ATP, a requirement for merozoite invasion [31]–[34]. Furthermore, this work will provide the foundation for future studies to identify new compounds that directly interfere with the invasion process.

## Materials and Methods

### Biochemicals

*Pfu* DNA polymerase and restriction enzymes were purchased from New England Biolabs (Ipswich, MA, USA). Ni^2+^-Sepharose™ High Performance chromatography resin was obtained from GE Healthcare Bio-Sciences AB (Uppsala, Sweden). Chemicals for gel electrophoresis were received from Biorad (Hercules, USA). The ATP- and ADP-analogues EDA-ATP ATTO-647N and EDA-ADP ATTO-647N were received from ATTO-TEC (Siegen, Germany). All other chemicals were at least of analytical grade and purchased from Sigma-Aldrich (Deisenhofen, Germany), Amersham Bioscience (Buckinghamshire, UK), BIOMOL (Hamburg, Germany) and Merck (Darmstadt, Germany).

### Cloning, Expression and Protein Purification

In order to obtain the truncated domains NBD94_444–547_, NBD94_566–663_ and NBD94_674–793_ of NBD94, the following primers were designed: forward primer 5′-AAC ATC GAA CCA TGG TAA TTC CAT-3′ and reverse primer 5′-CGC TTA TTT GGA GCT CTT ACG TTT-3′ for NBD94_444–547_; forward primer 5′- GAT CCA CCA TGG TAA AGG AAA-3′ and reverse primer 5′-GAT ATA TTA AAG AGC TCT TAT GTG TTC ATT T-3′ for NBD94_566–663_, and finally forward primer 5′-ATA AAG ATC CAT GGT ACA TTA TAT TAC TAG-3′ and reverse primer 5′-TTG ATT CGA GCT CTT ATA TTT TCG ATT-3′ for NBD94_674–793_. The genomic *P. yoelii* YM DNA was used as the template. In all the constructs the restriction sites *Nco*I and *Sac*I were incorporated. Following digestion with *Nco*I and *Sac*I, the PCR products were ligated into the pET9d1-His_3_ vector [54]. The pET9d-His_3_ vector, containing the respective gene, was then transformed into *E. coli* cells (strain BL21 (DE3)) and grown on 30 µg/ml kanamycin-containing Luria-Bertani (LB) agar-plates. To express NBD94_444–547_, NBD94_566–663_ and NBD94_674–793_, liquid cultures were shaken in LB medium containing kanamycin (30 µg/ml) for about 20 h at 37°C until an optical density of 0.6–0.7 (OD_600 _nm) was reached. To induce production of the recombinant proteins, the cultures were supplemented with isopropyl (thio)-β-D-galactoside (IPTG) to a final concentration of 1 mM. Following incubation for another 4 h at 37°C, the cells were harvested at 6 000×*g* for 20 min, 4°C. Selenomethionine containing protein was expressed in the same way except that methionine biosynthesis was inhibited by the growth conditions [55]. A 1∶1000 preculture in LB medium was used to inoculate 300 ml of M9 minimal medium supplemented with 0.4% glucose, 2 mM MgSO_4_, 30 µg/ml kanamycin, vitamins and trace elements. After overnight growth, the culture was diluted 1∶100 into 1 l of minimal medium. After an OD_600_ of 0.6 was reached, 100 mg/l DL-selenomethionine (Sigma), 100 mg/l lysine, threonine and phenylalanine and 50 mg/l leucine, isoleucine and valine were added as solids. IPTG (1 mM) was added after 15 min, and cells were grown for 3 h and harvested as described above. Cells were lysed on ice by sonication for 3×1 min in buffer A (50 mM Tris/HCl, pH 7.5, 200 mM NaCl, and 2 mM PMSF) for NBD94_444–547_, buffer B (50 mM Tris/HCl, pH 9.0, 200 mM NaCl, and 2 mM PMSF) for NBD94_566–663_ and buffer C (50 mM Tris/HCl, pH 7.5, 200 mM NaCl, 2 mM PMSF and 0.5 mM DTT) for NBD94_674–793_. The lysate was centrifuged at 10 000×*g* for 35 min. The supernatant was filtered (0.45 µm; Millipore) and passed over a Ni^2+^-NTA resin column to isolate NBD94_444–547_, NBD94_566–663_ and NBD94_674–793_, respectively. The His-tagged protein was allowed to bind to the matrix for 1.5 h at 4°C and eluted with an imidazole-gradient (25–300 mM) in buffer A, B and C, respectively. Fractions containing His-NBD94_444–547_, NBD94_566–663_ and NBD94_674–793_, were identified by SDS-PAGE [56], pooled and concentrated using Centricon YM-3 (3 kDa molecular mass cut off) spin concentrators (Millipore). The concentrated samples were applied on a size-exclusion column (Superdex™ 75 10/300 GL, GE Healthcare) using their respective buffers with additional 10 mM EDTA. The purity of all protein samples were analyzed by SDS-PAGE [56] and the gels were stained with Coomassie Brilliant Blue G250. Protein concentrations were determined by the bicinchoninic acid assay (BCA; Pierce, Rockford, IL., USA).

### Circular Dichroism (CD) Spectroscopy

Measurements of steady state CD spectra were carried out in the far UV-light (185–260 nm) using a CHIRASCAN spectropolarimeter (Applied Photophysics, UK). The CD spectroscopy measurements of NBD94_444–547_, NBD94_566–663_ and NBD94_674–793_ were performed in their corresponding buffers (see above) using a 60 µl quartz cell (Hellma) at 18°C with 1 nm step points and a protein concentration of 2 mg/ml for each recombinant protein. The spectrum for the buffer was subtracted from the spectrum of the protein. The ellipticity values were calculated by the average of triple determinations for each sample with a bandwidth of 1 nm from 185 to 260 nm (1 nm step points). The CD data were converted to mean residue ellipticity (Θ) in units of deg x dmol^−1^×aa^−1^ using the software Chirascan version 1.2.1 (Applied Photophysics). Baseline corrected spectra were used as input for computer methods to obtain predictions of secondary structure. In order to analyze the CD spectrum, the following algorithms were used: Varselec [57], Selcons [58], Contin [59], K2D [60]; all methods as incorporated into the program Dicroprot [61] and NeuralNet [62].

### Fluorescence Correlation Spectroscopy (FCS)

Fluorescence correlation spectroscopy was performed at 25°C on a LSM 510 Meta/ConfoCor 3 (Zeiss, Jena, Germany) using the ATP-analogue EDA-ATP ATTO-647N (ATTO-TEC, Siegen, Germany). The temperature was adjusted to 25°C in an incubation chamber (Zeiss). The 633 nm laser line of a HeNe633 laser was attenuated to 6 mW and focused into the aqueous solution by a water immersion objective (40×/1,2 W Korr UL-VIS-IR, Zeiss). The proteins were dissolved in the buffer, containing 50 mM Tris, pH 7.5, and 200 mM NaCl. FCS was measured in 15 µl droplets of the diluted fluorescent derivatives of ATP, which were placed on Nunc 8 well chambered cover glass. Before usage, the cover glasses were treated according to Hunke *et al.* (2007) [63]. Solutions of Cyanine 5 (Cy5) in pure water (*Fluka*) were used as references for the calibration of the confocal microscope. The following filter sets were used: MBS: HFT 514/633, EF1: BP 655–710, EF: None, DBS: None. Out-of-focus fluorescence was rejected by a 90 µm pinhole in the detection pathway. The confocal detection volume was calculated to be approximately 0.32 fl at λ = 655 nm at a numerical aperture NA of 1.2. Variable concentrations of NBD94_444–547_ protein solutions were mixed with MgCl_2_ and fluorescently labelled nucleotide, vortexed, spun down rapidly to assure the volume and placed the drop instantaneously onto the glass slip. The drop was incubated for 3 min, which was monitored by FCS. The fluorescence autocorrelation functions were determined by measurements of about 10 repetitions with 30 sec each. To analyze the autocorrelation functions of fluorescent nucleotides bound to protein, models with the diffusion time and the triplet state were used for fitting (FCS-LSM software, ConfoCor 3, Zeiss). The diffusion times of fluorescent nucleotides were measured independently and the determined values were kept fixed during the fitting of the FCS data. Based on this, the determination of the binding constants required only the calculation of the relative amounts of free nucleotides with the short diffusion time and of the bound nucleotides with the diffusion time of protein. The calculations of the bound fractions and the dissociation constants were done by the ConfoCor 3-software 4.2, Excel2003 and OriginPro 8 SR4.

In order to study the effect of NBD-Cl, NBD94_444–547_ solution, magnesium chloride and NBD-Cl were pre-incubated for 30 min at 8°C. After addition of EDA-ATP ATTO-647N the solution was incubated for further 10 min. The following filter sets were used: MBS: HFT 514/633; EF1: BP 655–710IR; EF: none; DBS: none; DBS1: plate; DBS3: mirror, 633 nm: 6% transmission. To analyze the autocorrelation functions, a standard model was used for fitting (FCS-LSM software, ConfoCor 3, Zeiss). The calculations were done by the ConfoCor 3-software 4.2, Microsoft Excel 2003, and Origin 7.5.

### Small Angle X-Ray Scattering Experiments

The synchrotron radiation X-ray scattering data for NBD94_444–547_ and NBD94_674–793_ were collected following standard procedures on the X33 SAXS camera [64], [65] of the EMBL Hamburg located on a bending magnet (sector D) on the storage ring DORIS III of the Deutsches Elektronen Synchrotron (DESY). A photon counting Pilatus 1 M pixel detector (67×420 mm^2^) was used at a sample - detector distance of 2.4 m covering the range of momentum transfer 0.1<s<4.5 nm^−1^ (s = 4p sin(q)/l, where q is the scattering angle and l = 0.15 nm is the X-ray wavelength). The s-axis was calibrated by the scattering pattern of Silver-behenate salt (d-spacing 5.84 nm). The scattering patterns from NBD94_444–547_ and NBD94_674–793_ were measured at 8.0 mg/ml, respectively. Protein samples were prepared in 50 mM Tris/HCl (pH 7.5), 200 mM NaCl and 1.25 mM DTT as radical quencher and injected automatically using the sample-changing robot for solution scattering experiments at the SAXS station X33 [66]. The data were normalized to the intensity of the incident beam; the scattering of the buffer was subtracted and the difference curves were scaled for concentration. All the data processing steps were performed using the program package PRIMUS [67]. The forward scattering *I(0)* and the radius of gyration *R_g_* were evaluated using the Guinier approximation [68]. The shape of NBD94_444–547_ and NBD94_674–793_ in solution was built by the program GASBOR [37] as described in [69].

### Crystallization of NBD94_566–663_

NBD94_566–663_ (10 mg/ml) was crystallized by vapor diffusion using 35% (v/v) 2-methyl-2,4-pentanediol as precipitant and acetate buffer of pH 4.5 (Wizard II Screen, Emerald BioSystems, Inc. USA) at 18°C. To avoid oxidation of selenomethionine during purification and crystallization, 1 mM tris-2-carboxyethyl-phosphine (TCEP) was added in the buffer. The crystal quality was improved by controlling the rate of vapor diffusion by the introduction of an oil barrier over the reservoir of a vapor-diffusion trial. The two different oils, paraffin and silicone, were applied as barriers, separately and also as mixtures of these two oils.

### Structure Determination

Crystals of selenomethionine (SeMet) substituted NBD94_566–663_ were prepared for MAD phasing. Initially, crystals were screened at beamline BL12B2 at SPring-8 using a Q4R detector. Native dataset and a complete MAD dataset from single crystals of SeMet incorporated NBD94_566–663_ were collected at 100 K on beamline BL26B2 at SPring-8 using a Mar CCD 225 detector. All the data were indexed, integrated and scaled using the program HKL2000 [38]. Selenium sites were identified and refined by the program SHELX [40], and density modification of the experimental map was performed with RESOLVE [46]. Manual model building and refinement of the structure was carried out in iterative cycles using COOT [47] and REFMAC5 [48]. The atomic coordinates and structure factors for the NBD94_566–663_ structure has been deposited in the Protein Data Bank under accession code 3HGF.

## Supporting Information

Figure S1 Fluorescence correlation spectroscopy studies of NBD94_444–547_. (A) Normalized autocorrelation functions of MgADP ATTO-647N obtained by increasing the quantity of NBD94_444–547_ (increased protein concentration from left to right). (B) Effect of increased NBD-Cl concentration of MgATP ATTO-647N bound to NBD94_444–547_ shown as normalized autocorrelation functions (increased effector concentration from right to left).
(0.53 MB DOC)

Figure S2 Crystal forms of NBD94_566–663_. Crystals of selenomethionine substituted NBD94_566–663_ (10 mg/ml) grown by vapor diffusion using 35% (v/v) 2-methyl-2,4-pentanediol as precipitant, acetate pH 4.5 and 1 mM tris-2-carboxyethyl-phosphine (A). The crystal size and quality has been improved by controlling the rate of vapour diffusion by the introduction of an oil barrier over the reservoir of a vapour-diffusion trial (B).
(5.00 MB DOC)

Figure S3 Simulated annealing omit map for chain A of the NBD94_566–663_ structure.
(0.46 MB DOC)

## References

[1] Rodriguez LE, Curtidor H, Uriquiza M, Cifuentes G, Reyes C (2008). Intimate molecular interactions of *P. falciparum* merozoite proteins involved in invasion of red blood cells and their implications for vaccine design.. Chem Rev.

[2] Huber M, Cabib E, Miller LH (1991). Malaria parasite chitinase and penetration of the mosquito peritrophic membrane.. Proc Natl Acad Sci USA.

[3] Meis JF, Croes H, Mons B, van Belkum A, Ponnudurai T (1992). Localization of circumsporozoite protein in the sporogonic stages of *Plasmodium vivax*.. Parasitol Res.

[4] Vanderberg JP (1974). Studies on the motility of Plasmodium sporozoite.. J Protozool.

[5] Golenda CF, Starkweather WH, Wirtz RA (1990). The distribution of circumsporozoite protein (CS) in *Anopheles stephensi* mosquitoes infected with *Plasmodium falciparum* malaria.. J Histochem Cytochem.

[6] Gaur D, Mayer DCG, Miller LH (2004). Parasite ligand-host receptor interactions during invasion of erythrocytes by *Plasmodium* merozoites.. J Parasitol.

[7] Preiser PR, Kaviratne M, Khan S, Bannister LH, Jarra W (2000). The apical organelles of malaria merozoites: host cell selection, invasion, host immunity and immune evasion.. Microbes Infect.

[8] Iyer J, Gruner AC, Renia L, Snounou G, Preiser PR (2007). Invasion of host cells by malaria parasites: a tale of two protein families.. Mol Microbiol.

[9] Cowman AF, Crabb BS (2006). Invasion of red blood cells by malaria parasites.. Cell.

[10] Duraisingh MT, Triglia T, Ralph SA, Rayner JC, Barnwell JW (2003). Phenotypic variation of *Plasmodium falciparum* merozoite proteins directs receptor targeting for invasion of human erythrocytes.. EMBO J.

[11] Galinski MR, Medina CC, Ingravallo P, Barnwell JW (1992). A reticulocyte-binding protein complex of *Plasmodium vivax* merozoites.. Cell.

[12] Grüner AC, Snounou G, Fuller K, Jarra W, Renia L (2004). The Py235 proteins: glimpses into the versatility of a malaria multigene family.. Microbes Infect.

[13] Ogun SA, Scott-Finnigan TJ, Narum DL, Holder AA (2000). *Plasmodium yoelii*: effects of red blood cell modification and antibodies on the binding characteristics of the 235-kDa rhoptry protein.. Exp Parasitol.

[14] Rayner JC, Vargas-Serrato E, Huber CS, Galinski MR, Barnwell JW (2001). A *Plasmodium falciparum* homologue of *Plasmodium vivax* reticulocyte binding protein (PvRBP1) defines a trypsin-resistant erythrocyte invasion pathway.. J Exp Med.

[15] Stubbs J, Simpson KM, Triglia T, Plouffe D, Tonkin CJ (2005). Molecular mechanism for switching of *P. falciparum* invasion pathways into human erythrocytes.. Science.

[16] Iyer J, Grüner AC, Rénia L, Snounou G, Preiser PR (2007). Invasion of host cells by malaria parasites: a tale of two protein families.. Mol Microbiol.

[17] Ogun SA, Holder AA (1994). *Plasmodium yoelii*: brefeldin A-sensitive processing of proteins targeted to the rhoptries.. Exp Parasitol.

[18] Freeman RR, Trejdosiewicz AJ, Cross GA (1980). Protective monoclonal antibodies recognising stage-specific merozoite antigens of a rodent malaria parasite.. Nature.

[19] Holder AA, Freeman, RR (1981). Immunization against blood-stage rodent malaria using purified parasite antigens.. Nature.

[20] Iyer JK, Amaladoss A, Ganesan S, Preiser PR (2007). Variable expression of the 235 kDa rhoptry protein of *Plasmodium yoelii* mediate host cell adaptation and immune evasion.. Mol Microbiol.

[21] Preiser PR, Jarra W, Capiod T, Snounou G (1999). A rhoptry-protein-associated mechanism of clonal phenotypic variation in rodent malaria.. Nature.

[22] Snounou G, Jarra W, Preiser PR (2000). Malaria multigene families: the price of chronicity.. Parasitol Today.

[23] Galinski MR, Barnwell JW (1996). *Plasmodium vivax*: Merozoites, invasion of reticulocytes and considerations for malaria vaccine development.. Parasitol Today.

[24] Ellsworth ML (2004). Red blood cell-derived ATP as a regulator of skeletal muscle perfusion.. Med Sci Sports Exerc.

[25] Ellsworth ML, Forrester T, Ellis CG, Dietrich HH (1995). The erythrocyte as a regulator of vascular tone.. Am J Physiol.

[26] Sprague RS, Ellsworth ML, Stephenson AH, Lonigro AJ (1996). ATP: the red blood cell link to NO and local control of the pulmonary circulation.. Am J Physiol.

[27] Ramalingam KJ, Hunke C, Gao X, Grüber G, Preiser RP (2008). ATP/ADP binding to a novel nucleotide binding domain of the reticulocyte-binding protein Py235 of *Plasmodium yoelii*.. J Biol Chem.

[28] Di Virgilio F, Chiozzi P, Ferrari D, Falzoni S, Sanz JM (2001). Nucleotide receptors: an emerging family of regulatory molecules in blood cells.. Blood.

[29] Osipchuk Y, Cahalan M (1992). Cell-to-cell spread of calcium signals mediated by ATP receptors in mast cells.. Nature.

[30] Sprague RS, Ellsworth ML, Stephenson AH, Lonigro AJ (1996). ATP: the red blood cell link to NO and local control of the pulmonary circulation.. Am J Physiol.

[31] Dluzewski AR, Rangachari K, Wilson RJ, Gratzer WB (1983). Properties of red cell ghost preparations susceptible to invasion by malaria parasites.. Parasitol.

[32] Dluzewski AR, Rangachari K, Wilson RJ, Gratzer WB (1983). A cytoplasmic requirement of red cells for invasion by malarial parasites.. Mol Biochem Parasitol.

[33] Olson JA, Kilejian A (1982). Involvement of spectrin and ATP in infection of resealed erythrocyte ghosts by the human malarial parasite, *Plasmodium falciparum*.. J Cell Biol.

[34] Rangachari K, Beaven GH, Nash GB, Clough B, Dluzewski AR (1989). A study of red cell membrane properties in relation to malarial invasion. Mol Biochem.. Parasitol.

[35] O'Donnell RA, Hackett F, Howell SA, Treeck M, Struck N (2006). Intramembrane proteolysis mediates shedding of a key adhesin during erythrocyte invasion by the malaria parasite.. J Cell Biol.

[36] Triglia T, Tham W-H, Hodder A, Cowman AF (2009). Reticulocyte binding protein homologues are key adhesins during erythrocyte invasion by Plasmodium falciparum.. Cell Microbiol. In press.

[37] Svergun DI, Petoukhov MV, Koch MHJ (2001). Determination of domain structure of proteins from X-ray solution scattering.. Biophys J.

[38] Otwinowski Z, Minor W (1997). Processing of X-ray Diffraction Data Collected in Oscillation Mode.. Methods Enzymol.

[39] Matthews BW (1968). Solvent content of protein crystals.. J Mol Biol.

[40] Sheldrick GM (2008). A short history of SHELX.. Acta Cryst.

[41] Terwillinger TC, Berendzen, J (1999). Automated MAD and MIR structure solution.. Acta Cryst.

[42] Brunger AT, Adams PD, Clore GM, Delano WL, Gros P (1998). Crystallography & NMR system: A new software suite for macromolecular structure determination.. Acta Cryst.

[43] Collaborative Computational Project, Number 4. (1994). The CCP4 suite: programs for protein crystallography.. Acta Cryst.

[44] Schneider TR, Sheldrick GM (2002). Substructure solution with SHELXD.. Acta Cryst.

[45] Sheldrick GM (2002). Macromolecular phasing with SHELXE.. Z Kristallogr.

[46] Terwilliger TC (2000). Maximum-likelihood density modification.. Acta Cryst.

[47] Emsley P, Cowtan K (2004). Coot: model-building tools for molecular graphics.. Acta Cryst.

[48] Murshudov GN, Vagin AA, Dodson EJ (1997). Refinement of macromolecular structures by the maximum-likelihood method.. Acta Cryst.

[49] Laskowski RA, MacArthur MW, Moss DS, Thornton JM (1993). PROCHECK: a program to check the stereochemical quality of protein structures.. J Appl Cryst.

[50] Sutton R, Ferguson SJ (1985). Tyrosine-311 of a beta chain is the essential residue specifically modified by 4-chloro-7-nitrobenzofurazan in bovine heart mitochondrial ATPase.. Eur J Biochem.

[51] Orriss GL, Leslie AG, Braig K, Walker JE (1998). Bovine F1-ATPase covalently inhibited with 4-chloro-7-nitrobenzofurazan: the structure provides further support for a rotary catalytic mechanism.. Structure.

[52] Hong S, Pedersen PL (2008). ATP synthase and the actions of inhibitors utilized to study its roles in human health, disease, and other scientific areas.. Microbiol Mol Biol Rev.

[53] Schäfer HJ, Coskun Ü, Eger O, Godovac-Zimmermann J, Wieczorek H (2001). 8-N(3)-3′-biotinyl-ATP, a novel monofunctional reagent: differences in the F(1)- and V(1)-ATPases by means of the ATP analogue.. Biochem Biophys Res Commun.

[54] Grüber G, Godovac-Zimmermann J, Link TA, Coskun U, Rizzo VF (2002). Expression, purification, and characterization of subunit E, an essential subunit of the vacuolar ATPase.. Biochem Biophys Res Comm.

[55] Schäfer I, Bailer SM, Düser MG, Börsch M, Ricardo AB (2006). Crystal structure of the archaeal A_1_A_O_ ATP synthase subunit B from *Methanosarcina mazei* Gö1: Implications of nucleotide-binding differences in the major A_1_A_O_ subunits A and B.. J Mol Biol.

[56] Laemmli UK (1970). Cleavage of structural proteins during the assembly of the head of bacteriophage T4.. Nature.

[57] Chan KM, Delfert D, Junger KD (1986). A direct colorimetric assay for Ca^2+^-stimulated ATPase activity.. Analytic Biochem.

[58] Manavalan P, Johnson WC (1987). Variable selection method improves the prediction of protein secondary structure from circular dichroism spectra.. Analytic Biochem.

[59] Sreerama N, Woody RW (1993). A self-consistent method for the analysis of protein secondary structure from circular dichroism.. Analytic Biochem.

[60] Provencher SW (1982). A constrained regularization method for inverting data represented by linear algebraic or integral equations.. Comput Phys Commun.

[61] Andrade MA, Chacon P, Merelo JJ, Moran F (1993). Evaluation of secondary structure of proteins from UV circular dichroism spectra using an unsupervised learning neural network.. Prot Engin.

[62] Deleage G, Geourjon C (1993). An interactive graphic program for calculating the secondary structure content of proteins from circular dichroism spectrum.. Comput Appl Biosci.

[63] Hunke C, Chen WJ, Schäfer HJ, Grüber G (2007). Cloning, purification, and nucleotide-binding traits of the catalytic subunit A of the V_1_V_O_ ATPase from *Aedes albopictus*.. Prot Exp Pur.

[64] Boulin CJ, Kempf R, Koch MHJ, McLaughlin SM (1986). Nuclear Instruments and Methods in Physics Research Section A: Accelerators, Spectrometers, Detectors and Associated Equipment.. Nucl Instrum Meth A.

[65] Roessle MW, Klaering R, Ristau U, Robrahn B, Jahn D (2007). Upgrade of the small angle X-ray scattering beamline X33 at the EMBL Hamburg.. J Appl Crystallogr.

[66] Round AR, Franke D, Moritz S, Huchler R, Fritsche M (2008). Automated sample-changing robot for solution scattering experiments at the EMBL Hamburg SAXS station X33. J Appl Cryst.

[67] Svergun DI (1993). A direct indirect method of small-angle scattering data treatment.. J Appl Crystallogr.

[68] Guinier A, Fournet G (1955). Small-angle Scattering of X-rays, Wiley, New York.

[69] Svergun, DI, Bećirević A, Schrempf H, Koch MHJ, Grüber, G (2000). Solution structure and conformational changes of the Streptomyces Chitin-Binding Protein (CHB1).. Biochemistry.

