# Supplementary figures and images for: Structural Determination of Functional Units of the Nucleotide Binding Domain (NBD94) of the Reticulocyte Binding Protein Py235 of Plasmodium yoelii
Source: PLoS One. 2010 Feb 10;5(2):e9146. doi: 10.1371/journal.pone.0009146 (PMC2818847; doi:10.1371/journal.pone.0009146)

| **(A)**  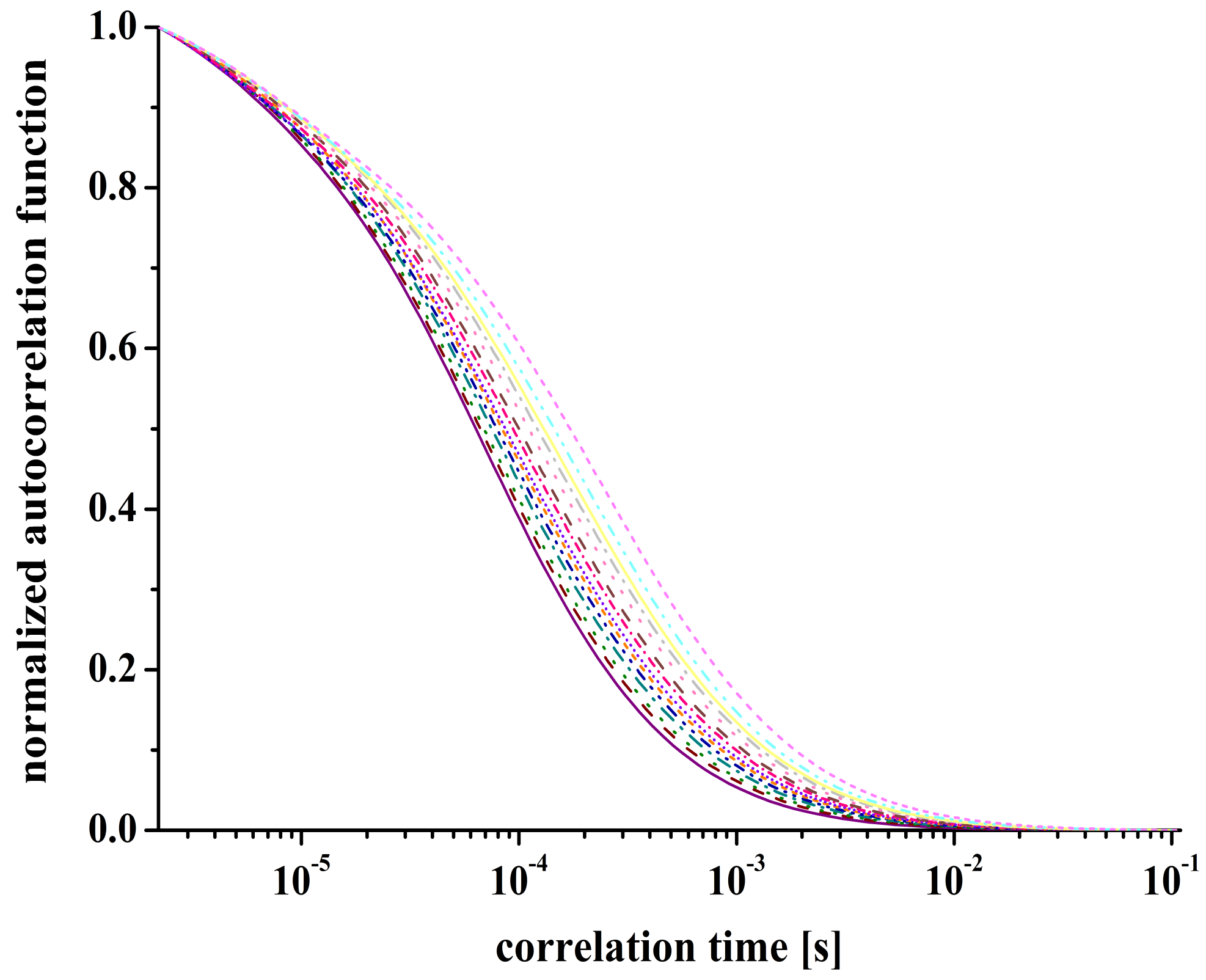 | **(B)**  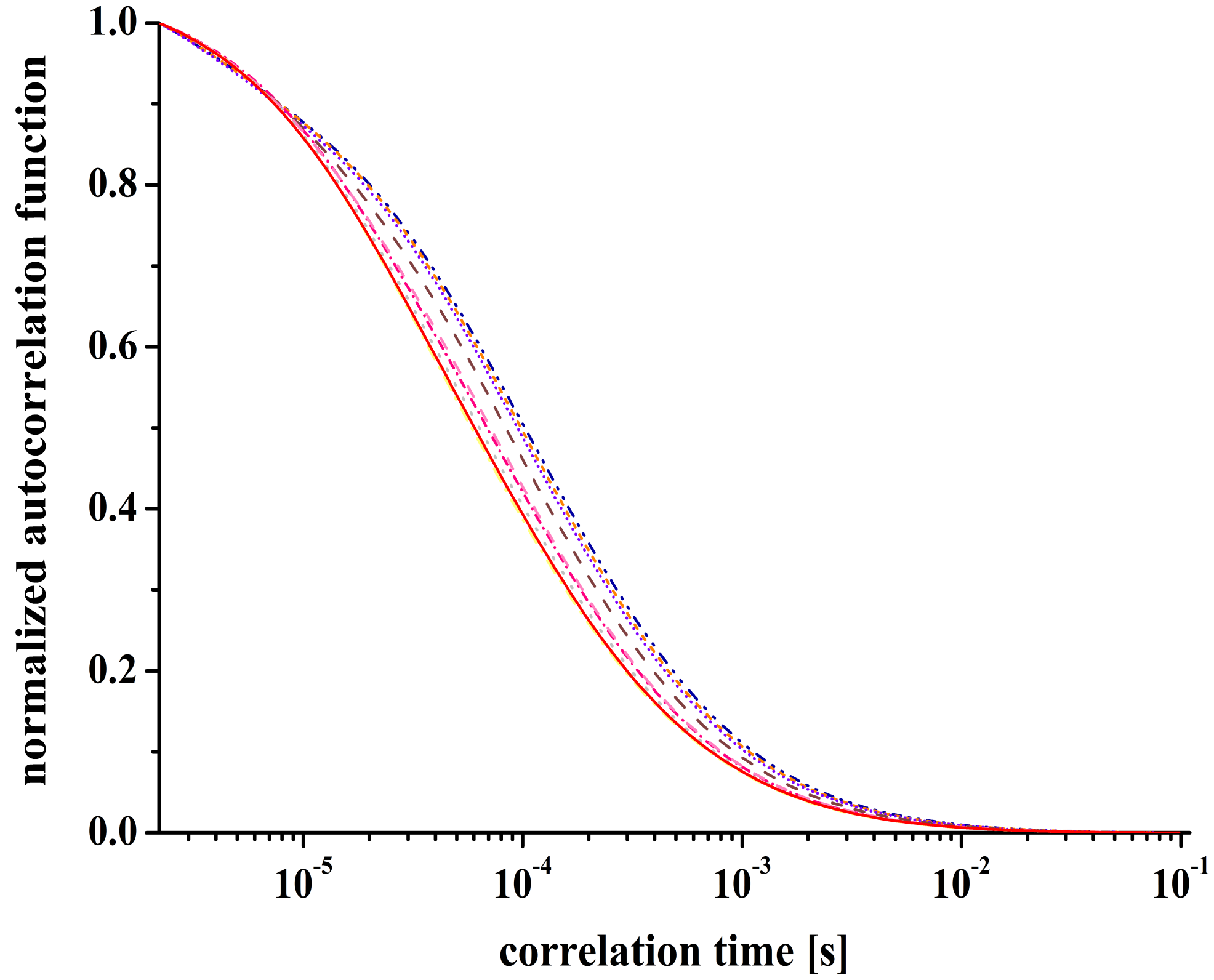 |
| --- | --- |

Supplement: Figure S1 — Fluorescence correlation spectroscopy studies of NBD94444–547. (A) Normalized autocorrelation functions of MgADP ATTO-647N obtained by increasing the quantity of NBD94444–547 (increased protein concentration from left to right). (B) Effect of increased NBD-Cl concentration of MgATP ATTO-647N bound to NBD94444–547 shown as normalized autocorrelation functions (increased effector concentration from right to left). (0.53 MB DOC) [file pone.0009146.s001.doc]

| (A) 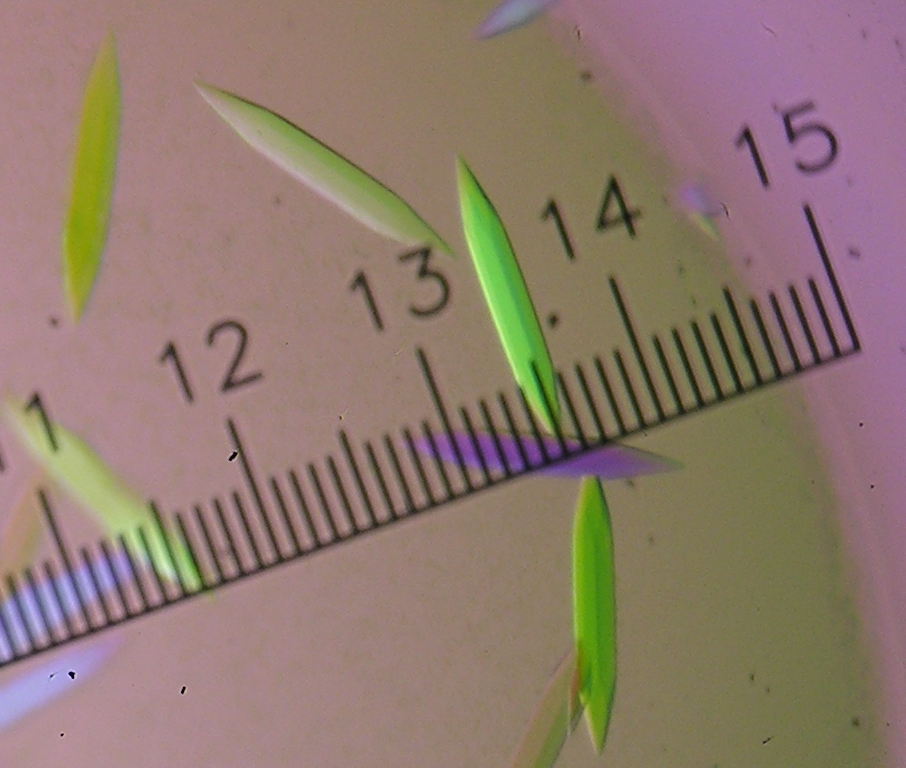 | (B) 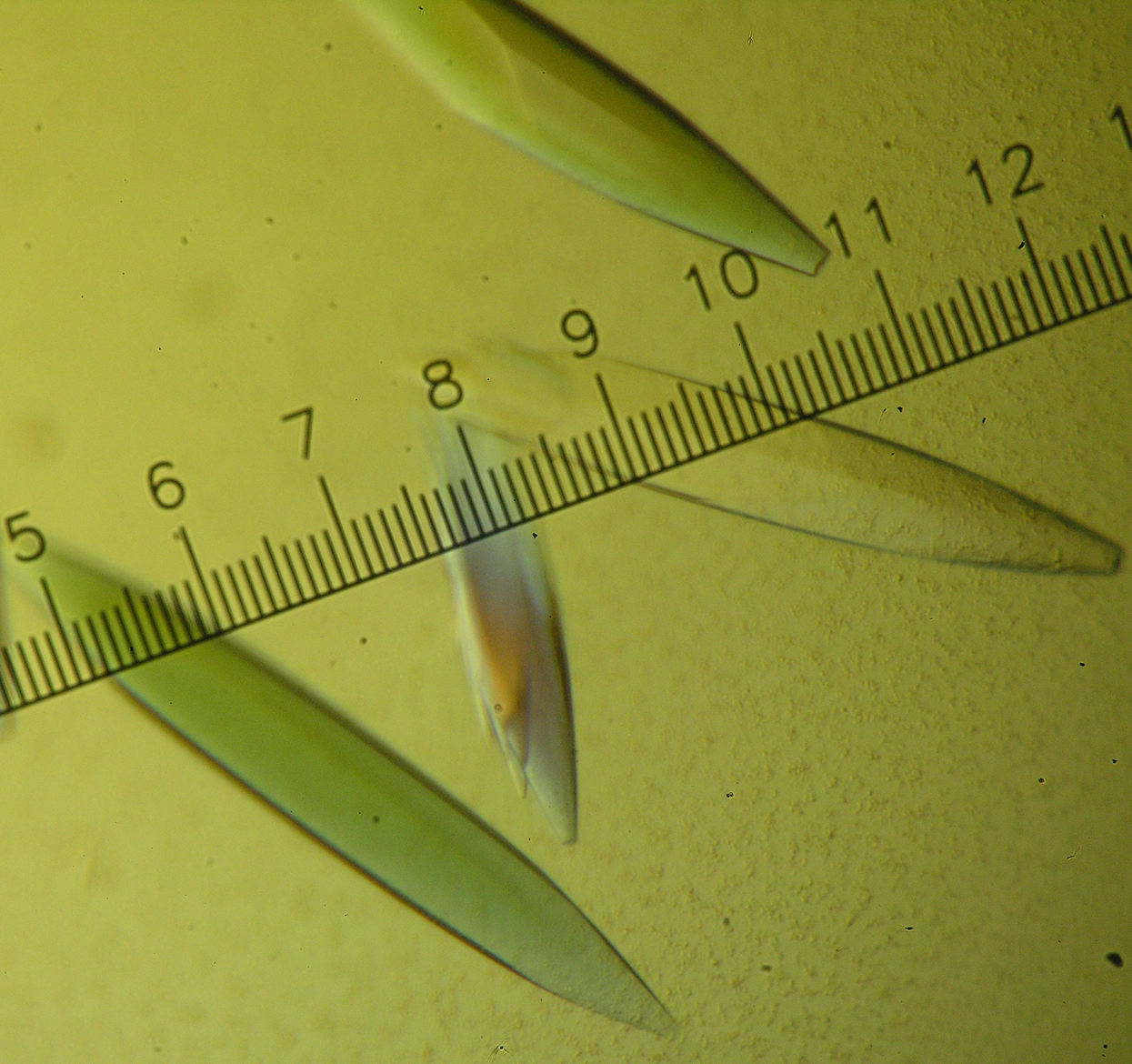 |
| --- | --- |

Supplement: Figure S2 — Crystal forms of NBD94566–663. Crystals of selenomethionine substituted NBD94566–663 (10 mg/ml) grown by vapor diffusion using 35% (v/v) 2-methyl-2,4-pentanediol as precipitant, acetate pH 4.5 and 1 mM tris-2-carboxyethyl-phosphine (A). The crystal size and quality has been improved by controlling the rate of vapour diffusion by the introduction of an oil barrier over the reservoir of a vapour-diffusion trial (B). (5.00 MB DOC) [file pone.0009146.s002.doc]

| 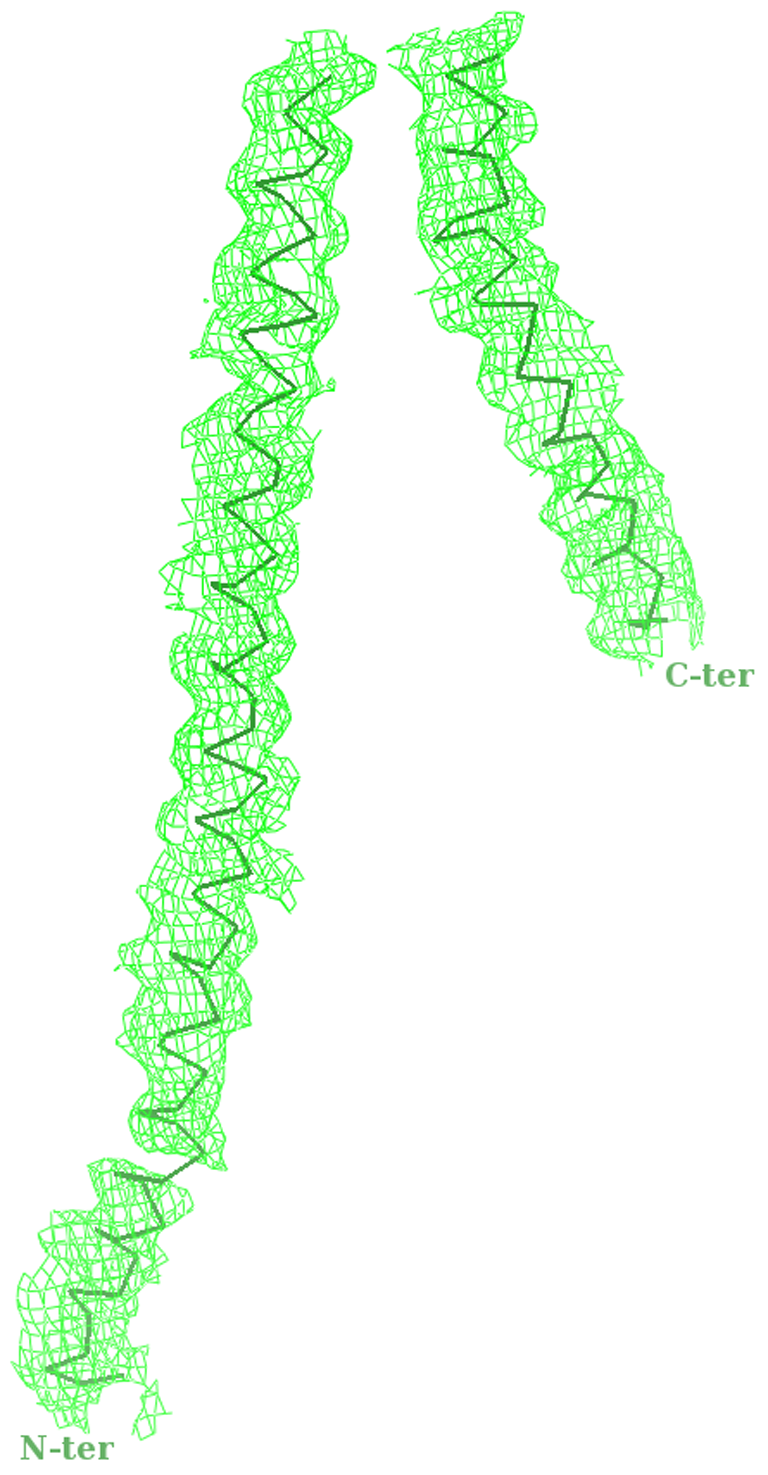 |
| --- |

Supplement: Figure S3 — Simulated annealing omit map for chain A of the NBD94566–663 structure. (0.46 MB DOC) [file pone.0009146.s003.doc]
